# Estrogen Induces Selective Transcription of Caveolin1 Variants in Human Breast Cancer through Estrogen Responsive Element-Dependent Mechanisms

**DOI:** 10.3390/ijms21175989

**Published:** 2020-08-20

**Authors:** Antonella Romano, Antonia Feola, Antonio Porcellini, Vincenzo Gigantino, Maurizio Di Bonito, Annabella Di Mauro, Rocco Caggiano, Raffaella Faraonio, Candida Zuchegna

**Affiliations:** 1 Department of Biology, University of Naples “Federico II”, 80126 Naples, Italy; antonella.romano2@unina.it (A.R.); antonia.feola@unina.it (A.F.); antonio.porcellini@unina.it (A.P.); candida.zuchegna@unina.it (C.Z.); 2 Pathology Unit, Istituto Nazionale Tumori Fondazione G. Pascale, 80131 Naples, Italy; gigantino.vincenzo@gmail.com (V.G.); m.dibonito@istitutotumori.na.it (M.D.B.); annab.dimauro@gmail.com (A.D.M.); 3 Department of Molecular Medicine and Medical Biotechnology, University of Naples “Federico II”, 80131 Naples, Italy; rocco.caggiano@unina.it

**Keywords:** Caveolin1, estrogen receptor, transcription, chromatin marks, epigenetic

## Abstract

The estrogen receptor (ER) signaling regulates numerous physiological processes mainly through activation of gene transcription (genomic pathways). Caveolin1 (CAV1) is a membrane-resident protein that behaves as platform to enable different signaling molecules and receptors for membrane-initiated pathways. CAV1 directly interacts with ERs and allows their localization on membrane with consequent activation of ER-non-genomic pathways. Loss of CAV1 function is a common feature of different types of cancers, including breast cancer. Two protein isoforms, CAV1α and CAV1β, derived from two alternative translation initiation sites, are commonly described for this gene. However, the exact transcriptional regulation underlying CAV1 expression pattern is poorly elucidated. In this study, we dissect the molecular mechanism involved in selective expression of CAV1β isoform, induced by estrogens and downregulated in breast cancer. Luciferase assays and Chromatin immunoprecipitation demonstrate that transcriptional activation is triggered by estrogen-responsive elements embedded in CAV1 intragenic regions and DNA-binding of estrogen-ER complexes. This regulatory control is dynamically established by local chromatin changes, as proved by the occurrence of histone H3 methylation/demethylation events and association of modifier proteins as well as modification of H3 acetylation status. Thus, we demonstrate for the first time, an estrogen-ERs-dependent regulatory circuit sustaining selective CAV1β expression.

## 1. Introduction

The estrogen receptor α and β (ERα, ER β) are members of the nuclear hormone receptor family, ligand-dependent transcription factors mediating signaling in estrogen-responsive cells [1]. Activation of the ERα-dependent pathways is largely dependent on 17β-estradiol (E2), the most active circulating sex hormone that also binds to ERβ. E2/ER signaling exerts pleiotropic effects by multiple mechanisms [2,3]. These are in part established by direct binding to gene promoters harboring estrogen-responsive element/s (ERE) and recruitment of specific coregulator/s that activate transcriptional machinery through histones posttranslational modifications and/or epigenetic DNA configurations [4,5,6]. Besides their role in gene expression regulation, ERs trigger non-genomic pathways initiated at membrane level through direct interaction with Caveolin1 (CAV1), the main structural protein of caveolae microdomains [7,8,9,10]. Caveolae are bulb-shaped invaginations of the plasma membrane enriched in cholesterol/glycosphingolipid with a distinct protein coat arising from a defined association of caveolins and cavins as structural elements. Caveolae are essential dynamic platforms for the activation and integration of a variety of membrane-initiated signaling pathways, including ER-dependent ones. Most of these signals lead to a rapid activation of protein kinases, such as Src, phosphatidylinositol 3-kinases (PI3K), protein kinase A (PKA) and protein kinase C (PKC) that trigger the membrane-initiated non-genomic signaling by estrogens [11,12,13]. ER-dependent pathways are required for a wide range of physiological processes including cell proliferation, formation of reproductive organs, maintenance of bone integrity and glucose homeostasis as well as protection of cardiovascular and brain tissues [14,15]. Deregulation of E2/ER signaling plays a critical role in the initiation and progression of target tissue malignancies, including breast cancer [16,17]. Although most of human breast tumors are ERα positive and anti-ER hormonal therapy represents the major therapeutic approach [18,19,20], the treatment of triple-negative breast cancers remains a challenge and still lacks a definite cure [19,20,21,22].

Caveolins comprise three distinct family members (CAV1, CAV2 and CAV3) that in human are encoded by the respective CAV genes [23]. Caveolin1 is expressed in most cell types, albeit with varying levels [23]. Because it is also found in other cellular sites including mitochondria, Golgi, endoplasmic reticulum and in secretory vesicles, CAV1 influences not only caveolae functions, but also modulates important intracellular processes [9,12,13,23]. Moreover, through its caveolin-scaffolding domain (CSD), CAV1 interacts and impacts the activities of different proteins, such as ERs discussed earlier and others involved in signal transduction (Src, EGFR, FYN), cell cycle control (HSP90AA1), metabolism (IRS1) and stresses (Nrf2, eNOS). For this reason, CAV1 dysfunction is associated with different type of human diseases including lipodystrophy, muscular dystrophies, pulmonary diseases and cancers, as demonstrated by numerous studies [23,24,25,26,27,28]. Growing evidence suggest that loss of CAV1 is a common feature of different types of cancers, including breast cancers. It has been reported, that in this context CAV1 acts as tumor suppressor [28,29,30,31].

Early studies demonstrated that CAV1 associates with ERs and that estrogens can contribute to CAV1 production [7,32,33,34], indicating a possible reciprocal functional control. Recently, we describe two major transcription start sites for CAV1, which differ for nuclear hormone dependence [35]. At the light of all these evidences, here we further characterize the molecular mechanism underling selective transcriptional regulation of CAV1 variants induced by estrogen signaling. Potentially these results could provide new molecular basis to improve the treatment and prognosis of triple-negative breast cancer.

## 2. Results

### 2.1. Expression of Caveolin1 Transcripts in Breast Cancer

**Figure 1:**
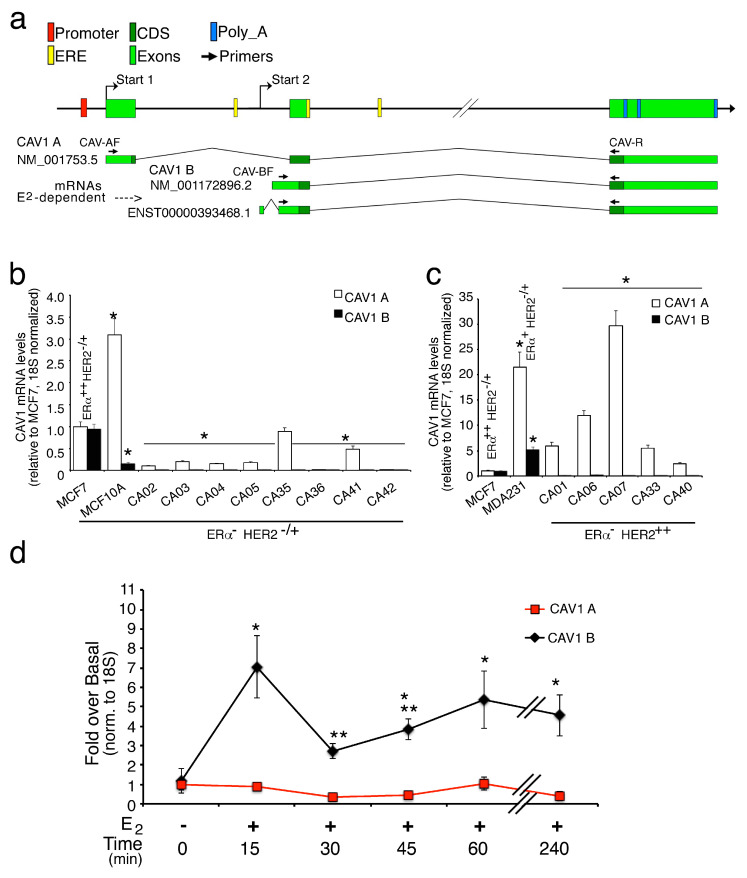
Human CAV1 transcript analyses in breast cancer tissues and cell lines. (**a**) Schematic representation of human CAV1 mRNA variants coding for Caveolin1 α (CAV1A) and β (CAV1 B) isoforms. E2-independent and E2-dependent transcription starts (Start 1 and Start 2, respectively) are indicated. The panel shows the transcript analyzed in this study. Arrows symbolize the primers used to detect CAV1 transcripts: The forward CAV-AF and CAV-BF primers detect the longer and shorter CAV1 transcripts, respectively (see M&M for the primer details); (**b**) mRNA levels of CAV1A and B transcripts, measured in MCF7, MCF10A (*n* = 6) and eight different ERα^-^ HER2^-/+^ cancer tissue samples. * *p* < 0.01 (matched pairs *t*-test), compared to MCF7; (**c**) CAV1A and B mRNA levels in MCF7, MDA231 (*n* = 6) and five different ERα^-^ HER2^++^ breast cancer tissues. Total mRNA was prepared from cells and tissue as described in M&M and analyzed by qPCR with the specific primers indicated in the panel a and normalized to 18S and relative to MCF-7. * *p* < 0.01 (matched pairs *t*-test), compared to MCF7 (**d**); E2 time-course expression analysis of CAV1A and B in MCF7 cells. All the transcripts were measured by RT-qPCR and normalized on 18S RNA levels. All values represent the mean relative mRNA levels setting as 1 the values obtained in absence of treatment (DMSO only). * *p* < 0.01 (matched pairs *t*-test), compared to E2-unstimulated sample; ** *p* < 0.01 (matched pairs *t*-test) compared to previous time point, *n* = 9.

Since Caveolin1 plays a critical role in breast cancer, and its down regulation increases tumor aggressiveness [31], we first explore the profiles of CAV1 mRNA variants in breast cell lines, like MCF7, that are ERα^++^ HER2^-/+^ cancer cells; MCF10A, that are triple negative normal cells; MDA231, that are triple negative cancer cells and in human breast tumors samples negative for ERα according to Subik K. et al. 2010 [36]. The immunophenotype classification on biopsies of this patient group is reported in Table 1. CAV1 protein is present in two main isoforms, namely CAV1α and CAV1β, of which the β variant lacks a region of 31 amino acids present at NH2-terminal of CAV1α [37], in fact CAV1 gene presents two major transcription starts (Start 1 and 2) originating the longer transcripts CAV1α (NM_001753.5) or CAV1β (NM_001172895.1) by alternative splicing and the shorter one CAV1β (NM_001172896.2, ENST00000393468.1) (Figure 1a). Hence, we first, tested the different transcript isoforms in MCF-7 cells using the specific primers indicated in Figure 1a. As both mRNA isoforms (CAV1A, longer; CAV1B, shorter) were present at similar level in MCF-7, this cell line was used as control (Figure 1b,c). In MCF10A the shorter CAV1B transcripts were significantly reduced and the longer 1A isoforms much more express (threefold); in MDA231, the expression of CAV1A and CAV1B transcripts were increased twenty and five times compared to control, respectively. Interestingly, when we tested the amounts of the aforementioned transcripts in tumor samples, a substantial reduction/not detectable level of CAV1B short isoforms was observed in all the tissues analyzed (Figure 1b,c). Moreover, within HER2 positive tumors, the CAV1A transcript levels show higher expression in comparison to the HER2 negatives (Figure 1b,c). These preliminary results suggest that loss of shorter transcripts of CAV1 is associated with tumor transformation into a more aggressive state and that these two different isoforms could exert opposite effects on tumor progression. As we previously shown, CAV1 gene reveals the presence of E2-dependent and E2-independent transcription start sites (TSS)s [35], so we measured the CAV1 transcripts levels in MCF7 cells exposed to E2. As shown in Figure 1d, the CAV1B short transcripts were strongly induced (sevenfold) after early stimulation (15 min); they were transiently reduced at 30 min and then stay higher than untreated cells during long E2 exposure. On the contrary, the E2-independent CAV1A transcripts, result unchanged under E2 treatments. Supplementary Figure S1a shows a dose-dependent increase in CAV1 and TFF1/pS2 (a positive E2-responsive gene) and CAV1 mRNAs levels, albeit CAV1 is less responsive than TFF1/pS2. For this reason, we decided to carry out the experiments with 50 nM of Estrogen since the basal transcription of a housekeeping gene, such as CAV1, can interfere with the study of the transcriptional response to lower doses of E2 (1–10 nM). Moreover, the treatment with the only vehicle (DMSO) does not alter the mRNA levels even after 60 min.

### 2.2. Functional Analyses of the Estrogen-Sensitive Regulatory Regions of the Human CAV1 Gene

**Figure 2:**
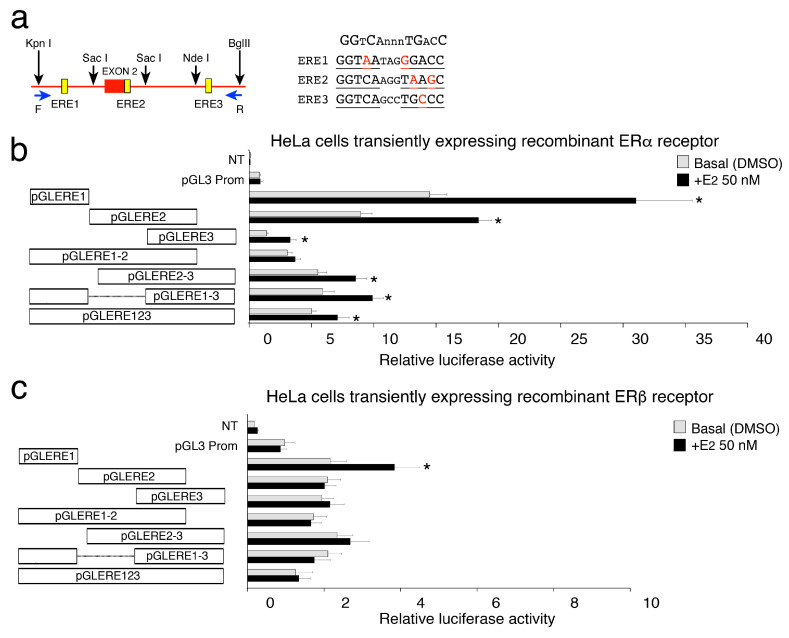
Transcriptional activity of CAV1 ERE sequences expressed as a fold increase in luciferase activity relative to pGL3 Prom. (**a**) Schematic representation of the CAV1 gene region containing the three ERE sequences studied, showed as yellow boxes. Forward and reverse primers used to amplify the region are shown (see blue arrows). Black arrows represent restriction enzymes sites used to obtain the different fragments. The sequences of three ERE sites (ERE 1, 2, 3) in CAV1 gene as well as the canonical ER response-element (on the top) are shown. Bases that do not correspond to the ER consensus sequence are in red. (**b**,**c**, on the left) The fragments indicated by white boxes were cloned upstream of Firefly luciferase promoter (pGL3–Promoter vector) to generate 7 luciferase reporter constructs. (**b**,**c**, on the right) Basal (DMSO only) and E2-induced luciferase activity of ERE containing constructs. HeLa cells were cotransfected with ERE constructs and ERα receptor (**b**) or ERβ receptor (**c**) expressing plasmids. PGL3 Promoter empty vector was used as control. To test ERE transactivation potential, 48 h after transfections, HeLa cells were synchronized for 6 h and then treated or not with 50 nM of E2 for 3 h. All the reported values represent the mean relative luciferase activities (RLU) of six experiments performed in triplicates, setting as 1 the values obtained with PGL3 * *p* < 0.01 (matched pairs *t*-test), compared to E2-unstimulated sample, *n* = 6. Data obtained in HeLa cells transfected with the control vector (not expressing the estrogen receptor) are shown in Supplementary Figure S1b.

Since it is unclear how cells can discriminate the selective transcription of CAV1A and CAV1B isoforms, we search for putative region/s with high homology with canonic ERE sequence through bioinformatic analysis. We identified an intragenic region containing three sequences that we called ERE1, ERE2 and ERE3 with 80% of homology with ERE consensus sequence (Figure 2a), of which one is located near the start of caveolin1β (Start 2) and the others downstream the second exon (Figure 1a).

To establish the functional role for these EREs, we have cotransfected HeLa cells with the pGL3–EREs constructs (see M&M and Figure 2a) and with or without estrogen receptor α-encoding vector for 48 h. Figure 2b shows that constructs harboring ERE1 or ERE2 regions significantly induce luciferase activity under estrogen treatment. For both ERE1 and ERE2 reporter constructs the basal luciferase activity was higher than that of the PGL3 empty promoter vector. The ERE3 construct exhibits a much lower intrinsic activity but retains a good transcriptional response to the estrogen. Instead no induction was seen when Hela cells was transfected whit the pGL3–EREs constructs alone (Figure S1b). Thus, the presence of a single ERE is sufficient to significantly induce luciferase activity upon E2 stimulation. When we analyzed the luciferase activity of larger regions, containing two or all three EREs, the basal activity was partially maintained, but the E2-sensitivity was strongly affected (Figure 2b). The higher distance from the transcription start, and/or the presence of genuine CAV1β start site/s, which could interfere with the reporter’s start site, can account for the lower E2-induced transcription shown by these constructs. Moreover, we explored the behavior of same constructs in the presence of estrogen receptor β and E2 treatment in HeLa cells. In these conditions, we observed a significant response only with constructs harboring the ERE1 region (Figure 2c).

Altogether, these results demonstrate that the intragenic regions studied contain functional estrogen responsive sequences, with strong E2-dependent activity when inserted immediately upstream the minimal luciferase promoter.

### 2.3. Estrogen Receptors are Differentially Recruited over Time on the Three ERE Regions

**Figure 3:**
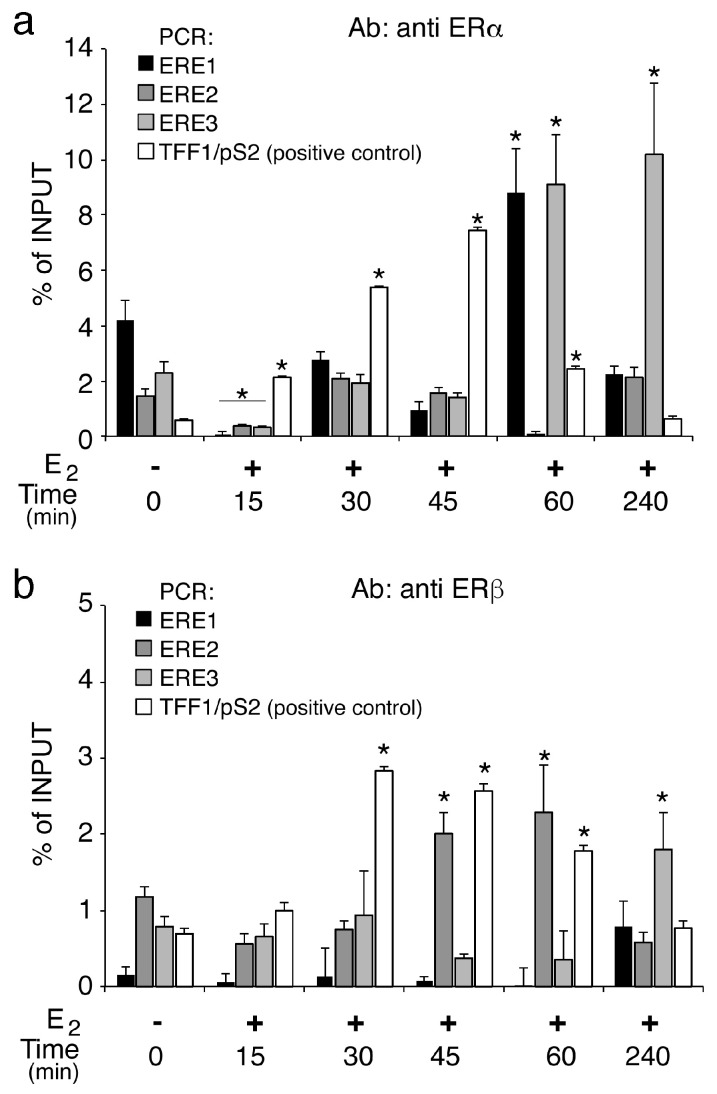
Differential recruitment of Estrogen receptors on the ERE regions. (**a**) ChIP analyses of ΕRα and ERβ (**b**) at the ΕRΕ1, ERE2 and ERE3 sites were performed on chromatin prepared from MCF7 cells upon E2 stimulation for a time course analysis as indicated. Recruitment of ΕRα and ERβ was determined on immunoprecipitated DNA by RT-qPCRs using specific oligonucleotide pairs designed for each ERE region. TFF1/pS2 ERE region was used as positive control [38] using primers described in M&M. The values reported were calculated as fold percentage of the amount of immunoprecipitated DNA relative to that present in total input chromatin. All data were normalized to IP controls. As negative control of an ERE-free region, exon 10 of the TSHR was amplified. qChIP data are presented as mean ±SD. * *p* < 0.01 (matched pairs *t*-test) compared to E2-unstimulated sample, *n* = 8 (*n* = 6 for TFF1/pS2).

To explore the mechanism underlying the transcription activation reported above, we analyzed the ER recruitment on the CAV1 and TFF1, as control region, ERE elements [38]. The genomic occupancy of ERα (Figure 3a) despite a significant decrease at 15 min, is induced between two-three-fold compared to untreated control, on ERE1 and ERE3 at 60 min and after 4 h it stabilizes on ERE3, unlike of control region (TFF1) where it is recluted in a time-dependent manner until 45 min. The recruitment of ERβ (Figure 3b) on the studied regions, demonstrates that this receptor compared to ERα is poorly associated with ERE1 and ERE3, but we found a significant increase (three-fold) at a later time point, between 45 and 60 min, on ERE2 (Figure 3b). Event in this case, on the control region, ERβ is recruited. The ERβ different dynamics could compensate the slight recruitment on of ERα on the ERE2. These results also support that the ERE regions used for functional studies are indeed capable of interacting with ERα and ERβ.

### 2.4. Histone H3K4 and H3K9 Methylation Marks Transcription Induced by Estrogen

**Figure 4:**
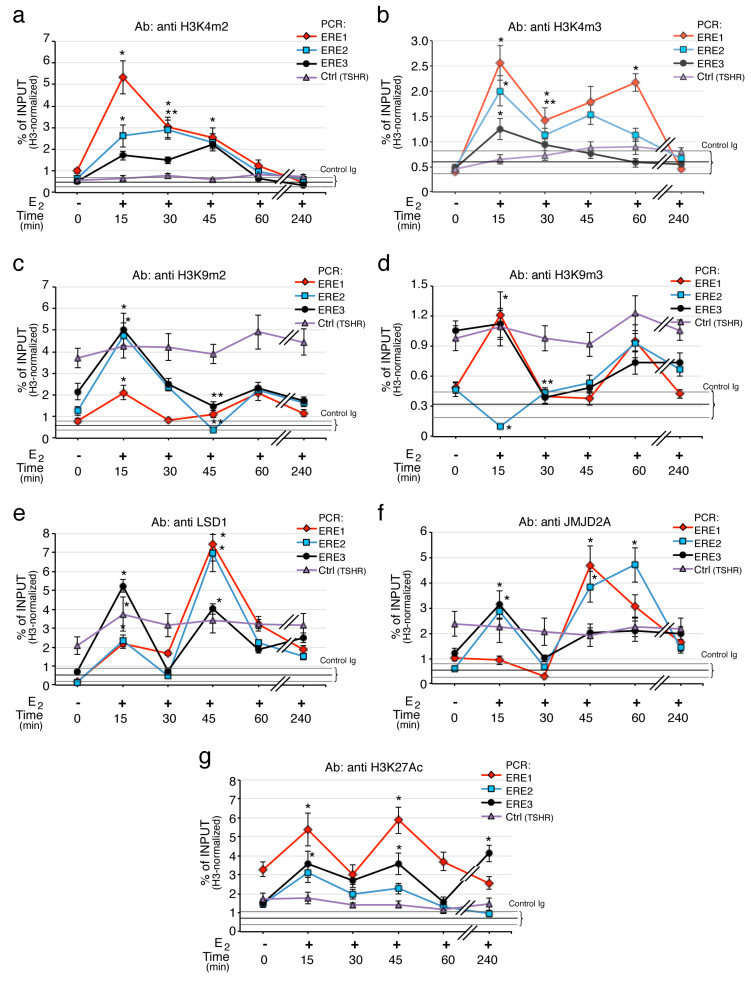
H3 post-translational changes and demethylases recruitment on CAV1 EREs. (**a**) Analysis of methylation status on H3K4 and H3K9 and acetylation of H3K27 at different ERE regions was performed by qChIP experiments using specific antibodies for (**a**) H3K4m2, (**b**) H3K4m3, (**c**) H3K9me2, (**d**) H3K9m3, (**g**) H3K27Ac. Panels (**e**,**f**) show the recruitment of LSD1 and JMJD2A, respectively. Enrichment was examined by ChIP-qPCR in MCF-7 cells upon E2 exposure at the indicated times. E2 nonresponsive TSHR gene was used as control region. Black, horizontal, line indicates the mean ± SD of the percent of input from a control ChIP carried out in parallel from the same lisate (Ab: non immune serum). H3 methylation levels were normalized to the input chromatin and H3 histone content. qChIP data are presented as mean of 3 independent experiments. * *p* < 0.01 (matched pairs *t*-test) compared to E2-unstimulated sample; ** *p* < 0.01 (matched pairs *t*-test) compared to previous time point, *n* = 6.

Gene transcription activated by ERs is predominantly coordinated by the methylation status of histones, occurring in particular on histone H3 [39]. Selective methylation of lysine 4 in histone H3 (H3K4) marks transcribed loci [40], whereas demethylation of lysine 9 (H3K9) in the same histone is associated with transcription silencing [41]. To explore this, we analyzed the profiles of H3K4 and H3K9 methylation in MCF-7 cells after E2 treatment. As a control, a genomic region of the E2 nonresponsive TSHR gene was used. The H3 methylation levels were normalized to the input chromatin and H3 histone content (Supplementary Figure S2a–d,f). The ChIP experiments indicate that estrogen induces selective transient histone H3 methylation and demethylation events on different CAV1 EREs regions. Specifically, shortly after E2 exposure (15−30 min), both activation marks on H3K4 (H3K4me2 and H3K4me3) accumulate at the ERE loci, in particular on ERE1 and ERE2. The di-methylated H3K4 rate on EREs progressively decreases across the treatment time, while tri-methylated H3K4 lasts up to 60 min on ERE1 (Figure 4a,b). On the contrary, the status of H3 methylation at the TSHR control gene was unaffected by E2 treatment.

The distribution pattern of histone H3 methylated on Lys9, shows that estrogens induce a transient three-fold increase of H3K9me2, mainly on ERE2 and ERE3 loci, with a peak occurring early at 15 min (Figure 4c). Meanwhile, H3K9m3 levels in response to E2, were significantly decreased on ERE2 and ERE3 regions and, intriguingly, it accumulated on ERE1 at 15 min (Figure 4d). After 60 min, all three regions were enriched in H3K9m3. In the same conditions, the amount of H3K4m1 undergoes few modifications (Supplementary Figure S2g), except for a strong decrease at 45 min on ERE1. The methylation changes after E2 stimulation are measurable despite a loss of H3 between 30 and 45 min indicating an opening of the chromatin after transcription initiation (Figure S1f). There are no significant changes in the histone code on a non-transcribed/ERE-less control region (exon 10 of the gene for the TSH receptor). Collectively, the ERE regions show discrete oscillations of methylation and demethylation involving H3K4 and H3K9, respectively. In addition, we noted that the location of the ERE (intron or exon) may modify the intensity of the methylation–demethylation profile. In fact, ERE1 that is placed in the intron region upstream to estrogen dependent transcriptional start (Figure 1a), shows the higher and significant epigenetic modifications.

Since Pezone et al. showed that the promoter region of CAV1 interacts with the intronic region containing the ERE regions [35], we also measured the histone code changes on the CAV1 promoter. The results (see Supplementary Figure S2e) show a methylation and demethylation cycle similar to that observed on the ERE1. These cyclical methylation–demethylation events were strikingly synchronous between promoter and ERE regions of CAV1 gene. In contrast, H3 methylation was unaffected by E2 at the TSHR site.

As H3K4me2 and H3K9me2 can be specifically demethylated by LSD1/KDM1A enzyme [42], while H3K9me3 and/or H3K9me2 demethylated by JMJD2A/KDM4A [43], we analyzed even their association profiles on the ERE regions upon E2 stimulation. Figure 4e shows that after 15 min of E2 exposure, LSD1 was strongly recruited on ERE3 region and also accumulates on ERE1; at 45−60 min the three ERE regions exhibited elevated levels of LSD1 compared to untreated controls. While we found that during the first 15 min, JMJD2A increases three times at ERE2 and ERE3 loci and then, within 30 to 60 min, it accumulates on ERE1 and ERE2 (Figure 4f). Of note, the kinetics of recruitment of LSD1 and JMJD2A parallels the kinetics of H3K4me2 depletion and H3K9me2/3 reduction observed on EREs.

To extend our analysis on the identified regions with possible enhancer function, we examined the profile of acetylation on Lys27 in H3 (H3K27Ac), as active chromatin epigenetic signature [44]. The kinetics of H3K27Ac shows that, following E2 exposure it accumulates at ERE1 and ERE3 sites, with two sharp peaks at 15 and 45 min (Figure 4g). The H3K27 acetylation well correlates with the peaks of tri-methylated H3K4 observed at 15 and 60 min on ERE1 in Figure 4b, thus indicating an active transcription state of CAV1 gene.

These results indicate that specific histone modifiers are recruited on EREs to modulate chromatin structure for selective transcriptional activation of caveolin1B.

## 3. Discussion

Loss of CAV1 by genetic manipulation [45,46] or functional defects [47,48] fosters dysplastic lesions and this is a common feature of different types of cancers, including breast cancers. In fact, results from different laboratories reported that in breast context CAV1 loss promotes tumor growth and metastasis [28,29,30,31]. However, caveolin1 plays complex roles in cancer and remains debated if it drives tumor suppression or cancer progression since in certain cancer contexts it seems to function as tumor promoter [34]. Thus, it is still unclear if altered expression of CAV1 is directly related to cancers or if aberrant signals drive anomalous CAV1 expression in different type of tumors [34].

The CAV1 gene encodes for longer alpha and shorter beta CAV1 (CAV1α, CAV1β) protein isoforms, originated by alternative splicing. Since we reported that this gene can also produce estrogen-dependent transcripts [35], to extend current knowledge on CAV1 transcriptional regulation we investigated the molecular basis of its dependence on estrogen-activated signals. We explored the transcript levels of CAV1A and CAV1B that are hormone-independent and hormone -dependent, respectively, in a small group of triple negative breast cancer samples and found that the mRNA levels of CAV1B are markedly reduced/not detectable in this type of tumors. This could indicate that transformation of these tumors to a more aggressive state may influence or be influenced by the loss of CAV1B transcripts. Additionally, within the group of HER2 positive tumors, CAV1A transcripts display elevated expression. Hence these findings offer clue for investigating whether CAV1A and CAV1B play different roles on breast tumor progression. It has been reported that CAV1 interacts with ERs and that estrogens can foster CAV1 production [7,32,33,34], suggesting a reciprocal functional control. In line with this, we demonstrate here, for the first time, that the CAV1B isoform is selectively induced under estrogen stimulation.

Estrogen-responsive genes are activated through the binding of estrogen receptor to consensus sequences known as ERE (estrogen responsive element) which could act as “enhancers”. Through bioinformatic analyses, we identified three intergenic sequences with elevate homology to the canonical ERE, which we named ERE1, ERE2, ERE3. Thus, our first aim was to explore the functional role of these putative estrogen responsive regions. Estrogen receptors work synergistically—and in order to regulate transcription—they could cooperatively bind multiple EREs located on the same side of the DNA helix [45,49]. By protein–protein interactions, ER dimers induce local conformational changes in chromatin status, and these allow accessibility to an adjacent ERE. Luciferase assays proved that these ERE regions under E2 stimulation, induce the expression of a reporter promoter gene. Interestingly, relative positions of ERE regions respect to the transcriptional start of CAV1 gene had a central role in the induction of estrogen-dependent transcription. In fact, ERE1 works well when it is near to the transcription start and worse when its distance increases or when it is in combination with the other sequences. This suggests that the activity of these sequences is specifically linked to a positional effect.

Gene expression mediated by regulatory elements is associated with epigenetic signatures involving, among others, specific histone modifications. In particular, the degree of methylation at lysine residues of histone H3 is an important predictive mark to study the transcription regulation. H3K4me3 marks actively transcribing genes [40], while H3K9 methylation is an indicator of silenced transcription and heterochromatin structure [41]. Our data highlighted that all ERE regions show discrete oscillations of H3K4 methylation and H3K9 de-methylation, ensuing proper transcription. Active enhancer-like regions are also marked by H3K27Ac signature [44] and we also found enrichment of histone H3K27 acetylation (H3K27ac) on the three EREs, with oscillatory kinetics.

Overall, our results demonstrate that CAV1 is an estrogen-dependent gene regulated via newly identified ERE-containing regions with enhancer-like activity. Estrogens drive selective transcription of caveolin1 beta encoding a protein that lacks the tyrosine-14 residue. As this latter is phosphorylated by non-receptor tyrosine kinases and is implicated in recruitment of cell migration signals, it can be speculated that this isoform possibly exerts negative effects on tumor progression. However, it remains to be proved.

## 4. Materials and Methods

### 4.1. RNA Extraction from Formalin-Fixed Paraffin-Embedded (FFPE) Breast Cancer Tissues and Cell Lines

Formalin-fixed paraffin-embedded tissues were from the “Biobanca Istituzionale dei Tessuti, Istituto Tumori Pascale”, Naples, Italy, as approved by Istituto Nazionale Tumori di Napoli, IRCCS G. Pascale, in the Resolution of the Extraordinary Commissioner; Number: 15, date: 20 January 2016. All patients signed a written informed consent. Samples included in this work were from patients with confirmed diagnosis of invasive ductal breast carcinoma (stage III). Total RNA was extracted twice from three consecutive sections of 1-μm-thickness of macro-dissected formalin-fixed paraffin-embedded (FFPE) tumor samples tissues using QIAGEN RNeasy FFPE Kit, according to manufacturer’s instructions (Qiagen Inc., Hilden, Germany). Cell total RNAs was extracted from an average of 3 million cells. Extraction was performed using the Acid guanidinium isothiocyanate–phenol–chloroform method. Briefly, 1 mL of a solution containing guanidine isothiocyanate (4 M), sodium citrate (25 mM, pH 7.0), N-lauroylsarcosine (0.5%) and β-mercaptoethanol (0.1 M) was added directly to the culture plates. The lysates were then complemented with 0.1 mL of sodium acetate (2 M, pH 4.2), 1 mL of acid phenol (saturated with 200-mM sodium acetate) and 0.2 mL of chloroform. Samples were centrifuged at 10,000× *g* for 30 min and the RNA present in the aqueous phase, precipitated with isopropanol. After centrifugation at 10,000× *g* for 20 min, RNA pellet was dissolved with UltraPure™ DEPC-treated Water After extraction, RNA yield and quality were determined with the NanoDrop 1000 Spectrophotometer. The A260/A280 ratio and A260/A230 of the RNA were used as indications of its purity. One microgram of total RNA was used for the cDNA synthesis in 20 µL final volume containing, 200 units of Superscript III Reverse Transcriptase (Invitrogen, Thermo Fisher, Carlsbad, CA, USA) and 1 µL of random primers (200 ng/µL). The reverse reaction was performed at 50 °C for 1 h and heat inactivated for 15 min at 70 °C. Quantitative Real Time PCR (RT-qPCR) reactions were performed on StepOnePlus™ Real-Time PCR System (Applied Biosystems™, Waltham, MA, USA) using PowerUp™ SYBR™ Green Master Mix (Applied Biosystems™). with cycle conditions as follows: 95 °C 10 min, 40 × (95 °C 15 s, 58 °C 90 s). The list of oligonucleotides (CAV-AF, CAV-BF, CAV-R) used is reported in Table 2.

### 4.2. Cell Lines and Transfections

Human breast cancer MCF-7 cells (ATCC^®^, Manassas, VA, USA, HTB-22™) and MDA-MB-231 (ATCC^®^ HTB-26™) were grown at 37 °C in 5% CO2 in Dulbecco’s modified Eagle’s medium (DMEM) supplemented with phenol red, L-glutamine (2 mM) and 10% fetal bovine serum (FBS, South America origin, Invitrogen). Non-tumorigenic breast MCF10-A (ATCC^®^ CRL-10,317™) cells were grown in a 1:1 mixture of DMEM and Ham’s F12 medium supplemented with 20-ng/mL epidermal growth factor, 100-ng/mL cholera toxin, 0.01-mg/mL insulin and 500-ng/mL hydrocortisone, 95%; horse serum, 5%. Cells were provided with fresh medium every 3 days. To evaluate the effect of estrogen challenge, cells were grown in phenol red-free DMEM containing 5% dextran–charcoal-stripped FBS for 3 days, before being challenged with 50 -nM in DMSO 17β-estradiol (E2) (CAS 50–28–2, Calbiochem, LaJolla, CA, USA) for different times according to the experimental needs. In all experiments, the control cells (E2-unstimulated samples/basal) were treated with vehicle alone (DMSO). HeLa cells (ATCC^®^ CCL-2™) were cultured at 37 °C in 5% CO2 in RPMI medium supplemented with 10% fetal bovine serum (Invitrogen), 1% penicillin–streptomycin and 2-mM glutamine. Transfections were performed using Lipofectamine 2000 Life Technologies (Rockville, MD, USA) following the manufacturer’s instructions. In all transfections, pEGFP C3 plasmid was included to determine and normalize transfection efficiency. All data used derived from experiments in which transfection efficiency was greater than 55%. Experiments with difference in transfection efficiency between different experimental points above 20% were discarded.

### 4.3. Plasmid Preparation and Luciferase Assays

The region of the human Caveolin 1 gene (1500 bp), encompassing the 3 estrogen-responsive DNA elements (EREs), was isolated by PCR from genomic human DNA using specific primer pairs containing KpnI and BglII sites in their sequence. The PCR product was cloned into the pGL3-Promoter vector, upstream from the Firefly luciferase promoter (Promega, Madison, WI, USA). We call this construct pGL3 ERE 1-2-3. The oligo adapters used to amplify the ERE 1-2-3 construct from the human genome by PCR are: Oligo Forward with the KpnI restriction site: 5′-GACGGTACCGCAGCGATAAAGGGAACATTCCAC-3′ Oligo Reverse with BglII restriction site is: 5′-GACAGATCTAGTCTGGCCAATAACCCAGTCCAA-3′. The other constructs that include different regions were obtained through enzymatic digestion (see Figure 2). pGL3 Cav ERE 1 digested by SacI and BglII; pGL3 Cav ERE 2 digested by NotI and XhoI; pGL3 Cav ERE 3 digested by KpnI and SacI; pGL3 Cav ERE 1-2 digested by BglII and NotI; pGL3 Cav ERE 2–3 digested by XhoI and NotI; pGL3 Cav ERE 1-3 digested with SacI and NotI. All clones were confirmed by enzymatic digestion and electrophoresis analysis. Luciferase reporter assays were performed as described [46,47], using the Luciferase Assays Kits 2.0 (#30,085–1, Biotium, Fremont, CA, USA) according to manufacturer’s instructions. Potential ERE regulatory elements were cloned into pGL3 Luciferase Reporter Vectors (Promega Corporation E1751) and cotransfected with ERα or ERβ receptor expressing plasmid into HeLa cells using lipofectamine method.

### 4.4. Chromatin Immuno-Precipitation (ChIP)

Chromatin immunoprecipitation was performed as previously described [48,49]. Briefly, cells were fixed for 10 min in formaldehyde 1% and the reaction was quenched by adding glycine 125 mM. Fixed cells were harvested and resuspended in Lysis Buffer (10-mM Tris-HCl pH 8.0, 10-mM NaCl, 0.2% NP40) containing 1X protease inhibitor cocktail (Roche Applied Science, Branford, CT, USA). The lysates were sonicated in order to have DNA fragments from 300 to 600 bp. Sonicated samples were centrifugated and supernatants diluted in ChIP buffer (1% Triton X-100, 2-mM EDTA, 150-mM NaCl, 20-mM Tris-HCl pH 8.0). An aliquot of sheared chromatin was further treated with proteinase K, extracted with 1 volume of phenol/chloroform/isoamyl alcohol and precipitated in LiCl 0.4 M/ethanol 75% to determine DNA concentration and shearing efficiency (input DNA). The ChIP reaction was set up as follows: the sheared chromatin was precleared for 2 h with 1 µg of non-immune IgG (Santa Cruz Biotechnology, Santa Cruz, CA, USA) and 20 µL of protein A/G PLUS-Agarose (Santa Cruz Biotechnology) saturated with salmon sperm (1 mg/mL). Precleared chromatin was divided in aliquots and incubated at 4 °C for 16 h with the specific antibody (for the codes, see below) or non-immune IgG (control Ig in Figure 4). The immunocomplexes were recovered by incubation for 3 h, at 4 °C with 20 µL of protein-A/G PLUS agarose, beads were washed according to manufacturer instructions, and immunoprecipitated DNA was recovered through phenol/chloroform/isoamyl alcohol extraction and ethanol precipitation and dissolved in TE buffer (10-mM Tris-HCl, 1-mM EDTA, pH 8.0). All data shown are the average of at least 6 independent experiments. The exact number for each experiment is indicated in the specific legend. Samples were subjected to RT-qPCR using specific primers reported in Table 2. Real Time-qPCRs were performed using StepOnePlus™ Real-Time PCR System (Applied Biosystems™) using PowerUp™ SYBR™ Green Master Mix (Applied Biosystems™) with cycle conditions as follows: 95 °C 10 min, 40 × (95 °C 45 s, 60 °C 90 s)

### 4.5. Antibodies

ERα ab32063 ((Abcam, Cambridge, UK); ERβ ab288 (Abcam); H3K4me1 ab8895 (Abcam); H3K4me2 ab32356 (Abcam); H3K4me3 ab1012 (Abcam); H3K9me2 ab1220 (Abcam); H3K9me3 ab8898 (Abcam); H3K27Ac ab4729 (Abcam); Total H3 ab1791 (Abcam); LSD1 ab17721 (Abcam); JMJD2A sc-271,210 (Santa Cruz Biotechnology); Normal rabbit IgG sc-2027 (Santa Cruz Biotechnology); Normal mouse IgG sc-2025 (Santa Cruz Biotechnology).

### 4.6. Statistical Analysis

All data are presented as mean ± standard deviation of at least two technical replicates. Data sets were analyzed statistically using the JMP Statistical Discovery™ software 6.03 by SAS (Statistical Analysis Software) and tested for normal distribution of variables using the Shapiro-Wilks test (“normal distribution fit” tool—JMP software). Statistical significance between groups was determined using Student’s *t*-test, one-way analysis of variance (ANOVA) or multivariate analysis of variance (MANOVA) for normally distributed values, Wilcoxon/Mann–Whitney U test or Kruskal–Wallis test for not normally distributed data. Two-tailed significance tests were performed with *p* < 0.05 considered significant. Statistical parameters for each experiment can be found within the corresponding figure legends.

## 5. Conclusions

The present study, albeit with limitations existing in verifying the biologic functions of E2-dependent CAV1 transcripts, highlights an important positive regulatory circuit that sustains selective CAV1 expression through estrogen responsive element-dependent mechanisms. Previous studies indicate that CAV1 could regulate ERs, but to the best of our knowledge, the finding that ERs regulate transcription of specific CAV1 isoforms has never been reported before. In light of the evidences reported here we thought that our data offer clues to better explore CAV1 isoforms in tumors, thus providing new opportunities to improve both treatment and prognosis of triple-negative breast cancer.

## Figures and Tables

**Table 1:** Characteristics of patients and tumor samples used in this study.

Sample	Age	HER2	ERα	PrR	Ki-67 (%)
01	64	++	−	−	45
02	73	−	−	−	70
03	39	−	−	−	60
04	87	−	−	−	15
05	65	−	−	−	60
06	56	++	−	−	50
07	46	++	−	−	70
33	30	++	−	−	50
35	41	−	−	−	40
36	67	−	−	−	85
40	42	++	−	−	45
41	69	−	−	−	65
42	43	−	−	−	30

ERα—estrogen receptor α; PrR—progesterone receptor.

**Table 2:** List of oligos used in this study.

Primer Name	Sequence	Locus
ChIP ERE 1 Fw	5′-GCGATAAAGGGAACATTCCAC-3′	CAV1
ChIP ERE 1 Rev	5′-CTGAGCGCTTCACCTGTTTC-3′	CAV1
ChIP ERE 2 Fw	5′-CTAAACACCTCAACGATGACG-3″	CAV1
ChIP ERE 2 Rev	5′-GTACACGGCAATGCTAAAG-3″	CAV1
ChIP ERE 3 Fw	5′-TGCACTTAGTGTGGAAAGC-3″	CAV1
ChIP ERE 3 Rev	5′-AATCTCACCAAGCACATCCTG-3′	CAV1
ChIP Promoter Fw	5′-AAACGTTCTCACTCGCTCTCTGCT-3′	CAV1
ChIP Promoter Rev	5′-GCGAGCAGAACAAACCTTTG-3′	CAV1
ChIP Fw	5′-ACCGAGACCCCTCTTGCTCT-3′	TSHR
ChIP Rev	5′-AGTTGCTAACAGTGATGAGAGGCT-3′	TSHR
ChIP ERE Fw	5′-ATGGGCTTCATGAGCTCCTTCC-3′	TFF1/pS2
ChIP ERE Rev	5′-GGAGTCTCCTCCAACCTGACCTTAAT-3′	TFF1/pS2
CAV-AF	5′-TTCCTCAGTTCCCTTAAAGCAC-3′	CAV1
CAV-BF	5′-GCCGCCCTCCCCGTCCTG-3′	CAV1
CAV-R	5′-CTTCTGGTTCTGCAATCACATC-3′	CAV1
TFF1 ERE Fw	5′-CCCTCCCAGTGTGCAAATA-3′	TFF1/pS2
TFF1 ERE Rev	5′-GATCCCTGCAGAAGTGTCTAAAA-3′	TFF1/pS2
RNA Fw	5′-GACCGATGTATATGCTTGCAGAGT-3′	18S
RNA Rev	5′-GGATCTGGAGTTAAACTGGTCCAG-3′	18S

## Supplementary Materials

Supplementary Materials can be found at https://www.mdpi.com/1422-0067/21/17/5989/s1.
