# Supplementary material for: Estrogen Induces Selective Transcription of Caveolin1 Variants in Human Breast Cancer through Estrogen Responsive Element-Dependent Mechanisms
Source: Int J Mol Sci. 2020 Aug 20;21(17):5989. doi: 10.3390/ijms21175989 (PMC7503496; doi:10.3390/ijms21175989)
Supplement: Supplementary file 1 [file ijms-21-05989-s001.pdf]

International Journal of
Molecular Sciences

MDPI

Estrogen induces selective transcription of Caveolin1 variants in human breast cancer through estrogen responsive element-dependent mechanisms

A. Romano, A. Feola, A. Porcellini, V. Gigantino, M. Di Bonito, A.Di Mauro, R. Caggiano, R. Faraonio *, C. Zuchegna

Supplementary Figure S1

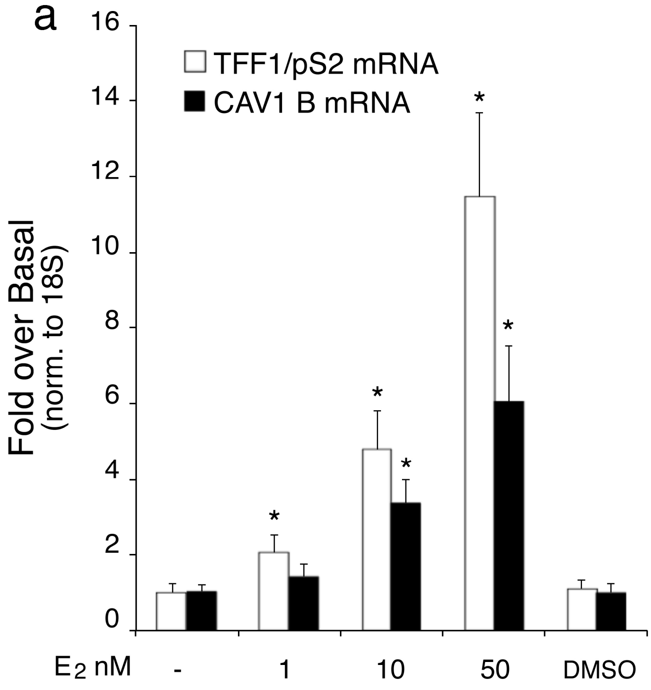

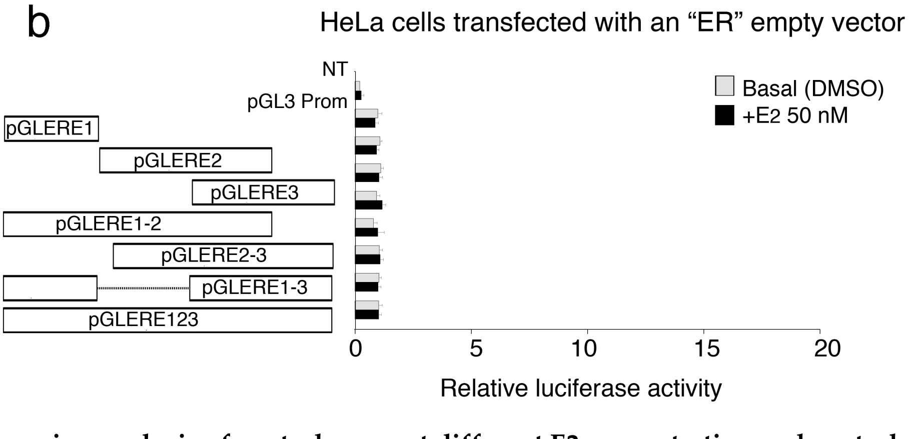

Figure S1. Expression analysis of control genes at different E2 concentration and control of transcriptional activity. (a) Expression analyses of *TFF1* and *CAV1B* genes were performed on MCF7 cells upon 1, 10 and 50 nM of E2 and only DMSO for 60 minutes. TFF1/pS2 mRNA were used as positive control. Total mRNA was prepared as described in M&M and analyzed by qPCR and normalized to 18S. * $p < 0.01$ (matched pairs t test), compared to E2-unstimulated sample, n= 6 **(b)** In Hela cells that do not express the estrogen receptors, pGL3 constructs do not express luciferase. The fragments indicated by white boxes were cloned upstream of Firefly luciferase promoter (pGL3-Promoter vector) to generate 7 luciferase reporter constructs. (on the right) Basal (DMSO only) and E2-induced luciferase activity of ERE containing constructs. HeLa cells were cotransfected with ERE constructs and pGL3 Promoter empty vector. To test ERE transactivation potential, 48 h after transfections, HeLa cells were synchronized for 6 h and then treated or not with 50 nM of E2 for 3 h.

International Journal of *Molecular Sciences*

MDPI

Supplementary Figure S2

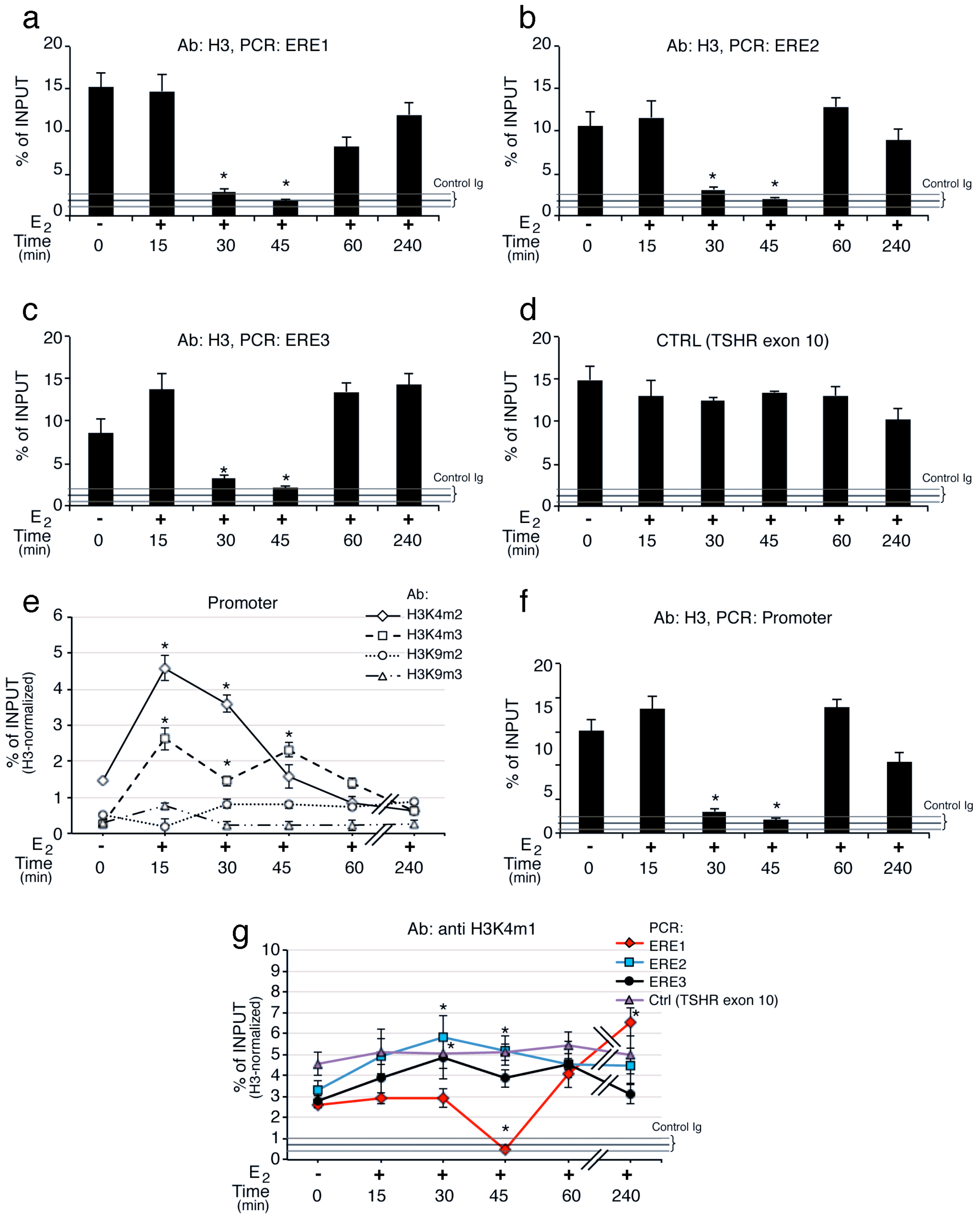

Figure S2. Total H3 levels analysis on CAV1 and TSHR gene, histone methylation on the promoter.

Total histone H3 enrichment levels on ERE1 (a), ERE2 (b), ERE3 (c), TSHR gene (d), methylation status on H3K4 and H3K9 at CAV1 promoter region (e), total histone H3 levels on CAV1 promoter (f),

International Journal of
Molecular Sciences

monomethylated H3K4 (H3K4m1) occupancy in the regions indicated of the CAV1 gene (g) are shown. All experiments are performed by ChIP-qPCR in MCF-7 cells exposed to E2 (50 nM) at the indicated times. The black, horizontal, line indicates the percent of input from control ChIP carried out in parallel from the same lisate (Ab: non immune serum). The H3 levels were normalized to the input chromatin. * $p<0.01$ (matched pairs t test) compared to E2-unstimulated sample.
